# Niche-Dependent Gene Expression Profile of Intratumoral Heterogeneous Ovarian Cancer Stem Cell Populations

**DOI:** 10.1371/journal.pone.0083651

**Published:** 2013-12-17

**Authors:** Sagi Abelson, Yeela Shamai, Liron Berger, Karl Skorecki, Maty Tzukerman

**Affiliations:** 1 Rappaport Faculty of Medicine and Research Institute, Technion-Israel Institute of Technology, Rambam Medical Center, Haifa, Israel; 2 Rambam Medical Center, Haifa, Israel; Cleveland Clinic, United States of America

## Abstract

Intratumoral heterogeneity challenges existing paradigms for anti-cancer therapy. We have previously demonstrated that the human embryonic stem cells (hESC)-derived cellular microenvironment in immunocompromised mice, enables functional distinction of heterogeneous tumor cells, including cells which do not grow into a tumor in a conventional direct tumor xenograft platform. We have identified and characterized six cancer cell subpopulations each clonally expanded from a single cell, derived from human ovarian clear cell carcinoma of a single tumor, to demonstrate striking intratumoral phenotypic heterogeneity that is dynamically dependent on the tumor growth microenvironment. These cancer cell subpopulations, characterized as cancer stem cell subpopulations, faithfully recapitulate the full spectrum of histological phenotypic heterogeneity known for human ovarian clear cell carcinoma. Each of the six subpopulations displays a different level of morphologic and tumorigenic differentiation wherein growth in the hESC-derived microenvironment favors growth of CD44+/aldehyde dehydrogenase positive pockets of self-renewing cells that sustain tumor growth through a process of tumorigenic differentiation into CD44-/aldehyde dehydrogenase negative derivatives. Strikingly, these derivative cells display microenvironment-dependent plasticity with the capacity to restore self-renewal markers and CD44 expression. In the current study, we delineate the distinct gene expression and epigenetic profiles of two such subpopulations, representing extremes of phenotypic heterogeneity in terms of niche-dependent self-renewal and tumorigenic differentiation. By combining Gene Set Enrichment, Gene Ontology and Pathway-focused array analyses with methylation status, we propose a suite of robust differences in tumor self-renewal and differentiation pathways that underlie the striking intratumoral phenotypic heterogeneity which characterize this and other solid tumor malignancies.

## Introduction

It is now widely appreciated that a single tumor is comprised of heterogeneous cell populations, each of which displays a diverse cellular morphology, phenotypic expression, tumor initiation capacities and inherent or acquired resistance to anti-cancer drugs. The recently published research describing intratumoral heterogeneity of colorectal cancer cellular and functional behavior, which are displayed by extensive variation in growth dynamics, persistence through serial xenograft passages and response to therapy further emphasizes the complexity of human tumors [1]. 

The aggressiveness and ingenuity of human cancers emanate mainly from such complex intratumoral heterogeneity, which in turn has been attributed to genetic and epigenetic changes coupled with adaptive responses to the tumor microenvironment. Accumulating evidence demonstrates that the model of 'cancer stem cells' (CSC) and the clonal evolution model, mutually contribute to intratumoral heterogeneity, as CSC themselves undergo clonal evolution [2-7]. The continuous accumulation of mutations generates heterogeneity of cells within a solid tumor and its metastases, and may reflect the process whereby certain subsets of tumor cells become more aggressive in the process of tumor progression. A crucial insight relates to understanding that the self-renewal capacity of cancer stem cells is not a durable state, but rather dynamic and niche-dependent. Tumor stroma interaction signals also regulate epithelial cancer cell plasticity via epithelial to mesenchymal transition programs (EMT) that in addition to facilitating invasion and metastasis of cancer cells, enable the conversion of non CSCs into CSCs and may influence the alteration of the tumor microenvironment by converting cancer cells into tumor supportive stroma [8-11]. As such, the complexity and plasticity of solid tumors emanates from the requirement of a supportive microenvironment that provides a compatible network of interactions between the heterogeneous cancer cells and various tumor-supporting cells [12-16]. Recruitment of tumor supporting multipotent mesenchymal stem cells coupled with extensive remodeling of adjacent tissues is an essential process for providing a microenvironment which supports cancer cell proliferation, migration and invasion [17,18]. The specific processes which have been most extensively studied include neoangiogenesis [19], attraction of inflammatory cells [20] and cancer associated fibroblasts [21-23] which together with the extracellular matrix (ECM) create the complexity of the tumor mass [24].

Xenotransplantation models for generating human tumors in immunodeficient mice are limited by differences between the murine and the human microenvironment, especially with respect to the niche-dependent properties of CSC self-renewal [25,26]. In many cases, this limitation leads to different read outs of distinct tumor subclones in xenotransplantation assays [5,21,27,28]. Accordingly, we have established and validated a tumor microenvironment model based on the potential of human embryonic stem cells (hESC) to generate teratomas in immunodeficient mice. This model has the ease of standard xenograft models, but the advantage that the tumor microenvironment is comprised of a wide variety of non-transformed differentiated tissues derived from all three germ layers and structures of human origin [29,30]. We have previously demonstrated that this *in vivo* model provides a preferential tumorigenesis microenvironment with the following features: a) enhanced tumor cell viability; b) prominent tumor cell invasion; c) tumor induced vasculogenesis; and d) relative protection from immunotoxin induced regression [29,30]. 

Ovarian clear cell carcinoma (OCCC), is characterized by striking intratumoral morphologic heterogeneity, including cells with features of advanced ovarian structural variation on the one hand, and cells with features of tumorigenic differentiation (e.g. invasion, proliferation) and corresponding cell surface and intracellular marker heterogeneity [31-35]. We have isolated and characterized six different cancer cell subpopulations (CCSPs) from a tumor of a single patient, and demonstrated niche dependent tumorigenic capacities and histological phenotypes when grown within the hESC-derived teratoma tissue, which cumulatively recapitulate the full spectrum of tumor heterogeneity [36]. The six CCSPs were characterized as ovarian CSC by virtue of functional and phenotypic expression of CD44+CD24+EpCAM+ and ALDH1 activity [5,36]. In addition, these six distinct subpopulations have been functionally characterized as displaying the key stem cell properties of both self-renewal and tumorigenic differentiation capacities in a niche dependent manner. In the murine niche, there is limited support for self-renewal of CSC, together with a high rate of tumorigenic differentiation, while the hESC-derived niche more prominently maintains CSC self-renewal as opposed to differentiation. Thus, the hESC-based model provides a crucial *in vivo* platform in view of the essential role of CSC self-renewal in the resistance to anti-cancer therapies and in rendering intratumoral heterogeneity amenable to biological analysis as well as anticancer therapy testing [5]. Furthermore, it was recently demonstrated that the hESC-based model express *bona fide* human tumor blood vessels and enhance tumor engraftment rate by primary human ovarian cancer stem-like cells (CSC) [37]. Accordingly, we aimed to examine the respective gene expression profiles of two different OCCC-derived CCSPs representing the extreme ends of the spectrum in terms of niche-dependent self-renewal versus tumorigenic differentiation. The results obtained highlight pathways governing intratumoral heterogeneity at the gene expression and epigenetic levels, and the important contribution of the tumor microenvironment to this heterogeneity. 

## Materials and Methods

### Derivation of ovarian cancer cell subpopulations

Collection of ascites fluid was performed with a written informed consent of a 64 year old patient diagnosed with stage IV Ovarian Clear Cell Carcinoma and the protocol was approved by the institutional Ethics Review Committee of the Rambam Medical Center. Six different cancer cell subpopulations, clonally expanded from a single cell, including CCSP C12 and C13, were derived from the malignant ovarian ascites and propagated in culture as previously described [5,36]. Clonality assays using the HUMARA method [38] and Forensic STR analysis indicated a monoclonal origin for all the six CCSPs examined (data not shown). Nevertheless, karyotype analysis of metaphase chromosomes extracted from each cancer cell subpopulation, spread on slides and analyzed by Spectral-Karyotyping (SKY), demonstrated a high level of chromosomal changes and variations among the CCSPs (data not shown). It should be noted that although maintained in culture for more than 6 years, cell cultures are repeatedly initiated from frozen stocks every 3-4 months, and the CCSPs durably and consistently maintain the "*bona fide*" ovarian cancer characteristics, CSC characteristics and xenografted tumor histological phenotype [5,36]. 

### Animals, teratoma, tumor formation and tissue handling

SCID/beige mice were purchased from Harlan Laboratories Ltd., Jerusalem, Israel. The mice were housed and maintained under specific pathogen-free conditions as instructed by the Committee for Oversight of Animal Experimentation at the Technion - Israel Institute of Technology, Haifa, Israel. This was approved by the Institutional Animal Care and Use Committee of the Technion (Protocol # IL-026-02-12). Mice were observed for teratoma and tumor formation once a week and sacrificed using CO_2_ inhalation. For teratoma formation, undifferentiated hESC from clone H9.1 (46XX), were injected into the mice hindlimb musculature (~5x10^6^ cells per injection) [29]. At 7-8 weeks following initial injection of the hESC, 4X10^6^ cancer cells were injected into the teratoma (i.t tumors) and were allowed to grow for an additional 20 to 60 days. Control tumors derived from direct injection (i.m tumors) of 4x10^6^ cells into the hindlimb musculature were harvested at 20 to 60 days following injection.

### Gene expression studies

The expression studies were conducted with three independent sample sets using the Illumina HumanWG-6 V3.0 expression beadchip Array. The scanned images were analyzed using Illumina's GenomeStudio V.2009.1 software (Illumina Inc. San Diego, CA, USA) for extraction, and quality control. The raw data obtained from the three independent microarray experiments were imported into genomics software (Genomics Suite; Partek Inc., St Louis, MO), normalized, and a removal of any non-biological, "batch effects", which may interfere with the results performed. The gene expression signals were transformed by computing the base two logarithms. Genes that their given transcript was not expressed above the background defined by negative control probes of the Illumina beadchip in all samples were filtered out. The data have been deposited in the National Center for Biotechnology Information GEO7, and are accessible through GEO Series accession GSE43208.

### Differential expression of single genes

Genes which are differentially regulated in each group, were selected based on their fold change. The average of the normalized genes signals among the various groups was compared and Student’s t-test was used for assessing the statistical significance of the fold difference between them. Genes were selected based on fold change (>2), and corresponding statistical significant p-value (<0.05). To reduce the chance of false positives, genes that did not follow the 2 fold threshold in each one of the 3 independent gene expression arrays were omitted.

### Gene Set Enrichment analyses (GSEA)

GSEA was performed using the JAVA-based software [39]. The Leading Edge Analysis tool (LEA) was used to extract the core gene members of the enriched gene sets in order to create lists of genes that make the greatest contribution to the corresponding enrichment scores (ES) obtained for either C12 i.m, C12 i.t, C13 i.m, and C13 i.t class distinctions. A conservative threshold of False Detection Rate (FDR) <0.05 and p-value<0.05 was determined after 1,000 random permutations. Only leading edge genes that were shared by at least 5 statistically enriched gene sets were used subsequently for the Gene Ontology analyses.

### Gene Ontology (GO) analysis

The Gorilla web-based tool (http://cbl-gorilla.cs.technion.ac.il) which uses ranked gene lists was used in order to identify enriched GO annotations [40]. As target list inputs, we used the core gene members of GSEA LEA tool correlates to C12 or C13 derived tumors that developed intra-muscular (i.m) or intra-teratoma (i.t). The four target lists were used separately, while a list which represents all the genes tested in the Illumina gene expression platform served as the background for each one of the analyses. A conservative threshold p-value<0.005 was chosen following Bonferroni correction for multiple comparisons.

### Restrictive analysis criteria

1) Genes that did not follow the 2 fold threshold in each one of the 3 independent gene expression arrays were omitted (highly restrictive criteria). 2) In the GSEA, conservative nominal p-value <0.05 (NOM p) and FDR q-value <0.05 were chosen 3) LEA was used. 4) In the GO enrichment analysis, cutoff of p-value<0.005 was chosen.

### Pathway-Focused Arrays

The human Wnt, Sonic Hedgehog, and Notch signaling pathway RT^2^ Profiler PCR arrays (SuperArray Bioscience, Frederick, USA) were used according to manufacturer’s instructions with 1 µg of RNA as starting material in each array. Analysis of the data was performed with SABiosciences web-based PCR Array Data Analysis software (http://sabiosciences.com/pcrarraydataanalysis.php) and Ingenuity Pathway Analysis (IPA) software (Ingenuity Systems, Redwood City, CA).

**RNA extraction from laser micro-dissected tumor samples, and from *invitro* growing cells, qRT-PCR analyses and Bisulfite sequencing** are described in Materials and Methods S1. Genes specific primers used for qRT-PCR and bisulfite analyses are provided in Tables S2 and S3.

## Results

### Expression profiles of OCCC-derived heterogeneous cancer cells *in vitro* and *in vivo*

**Figure 1:**
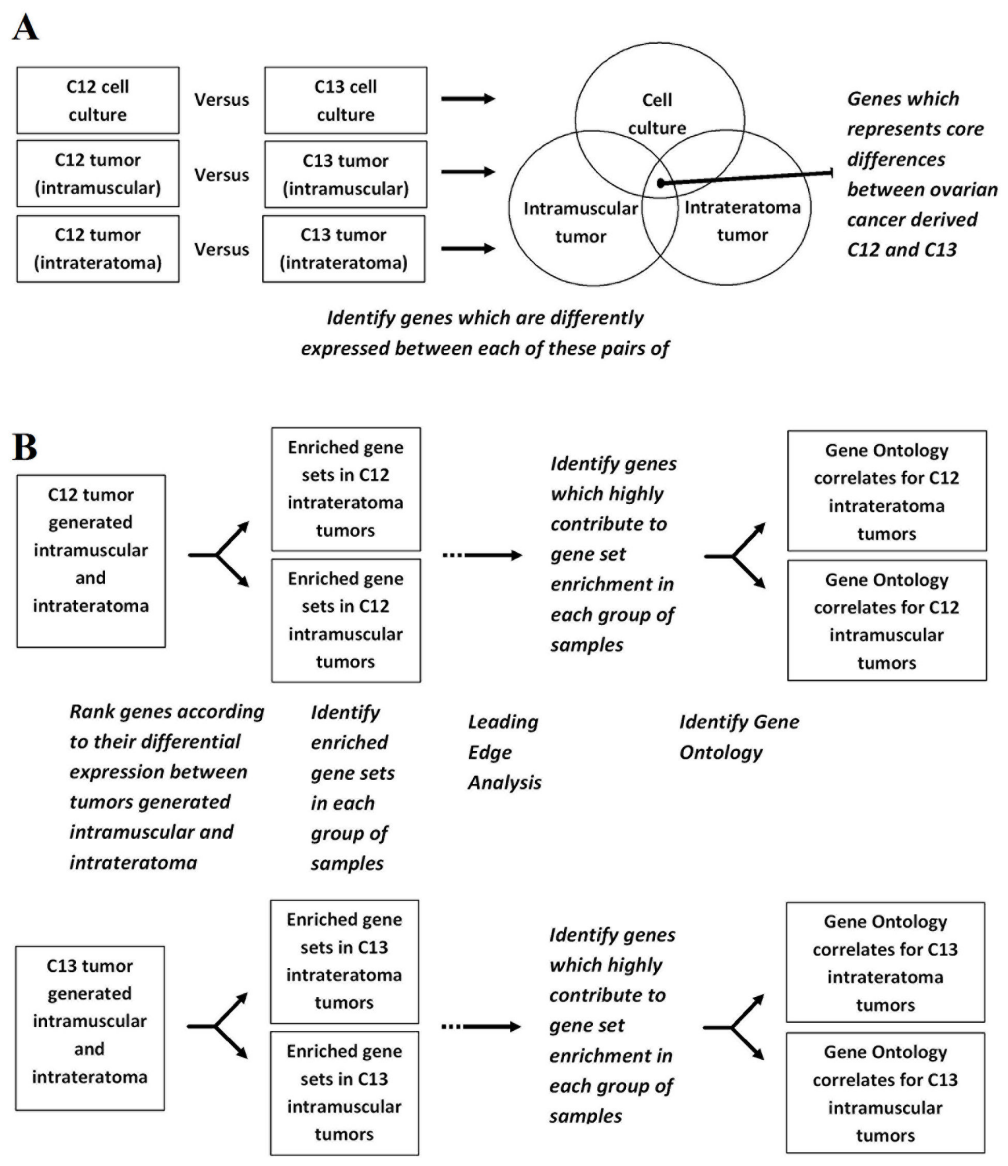
Data management workflow for gene expression profiling process. *A*, Workflow of the analyses performed for gene expression profiling of cancer cell subpopulations (CCSPs) C12 and C13 *in* *vitro* and *in* *vivo*. *B*, Identification of differentially expressed genes between C12 and C13 *in* *vitro* grown cells and between tumors generated intramuscular (i.m) and intrateratoma (i.t), and Gene Ontology annotations which correlate with tumors generated i.m and i.t.

**Figure 2:**
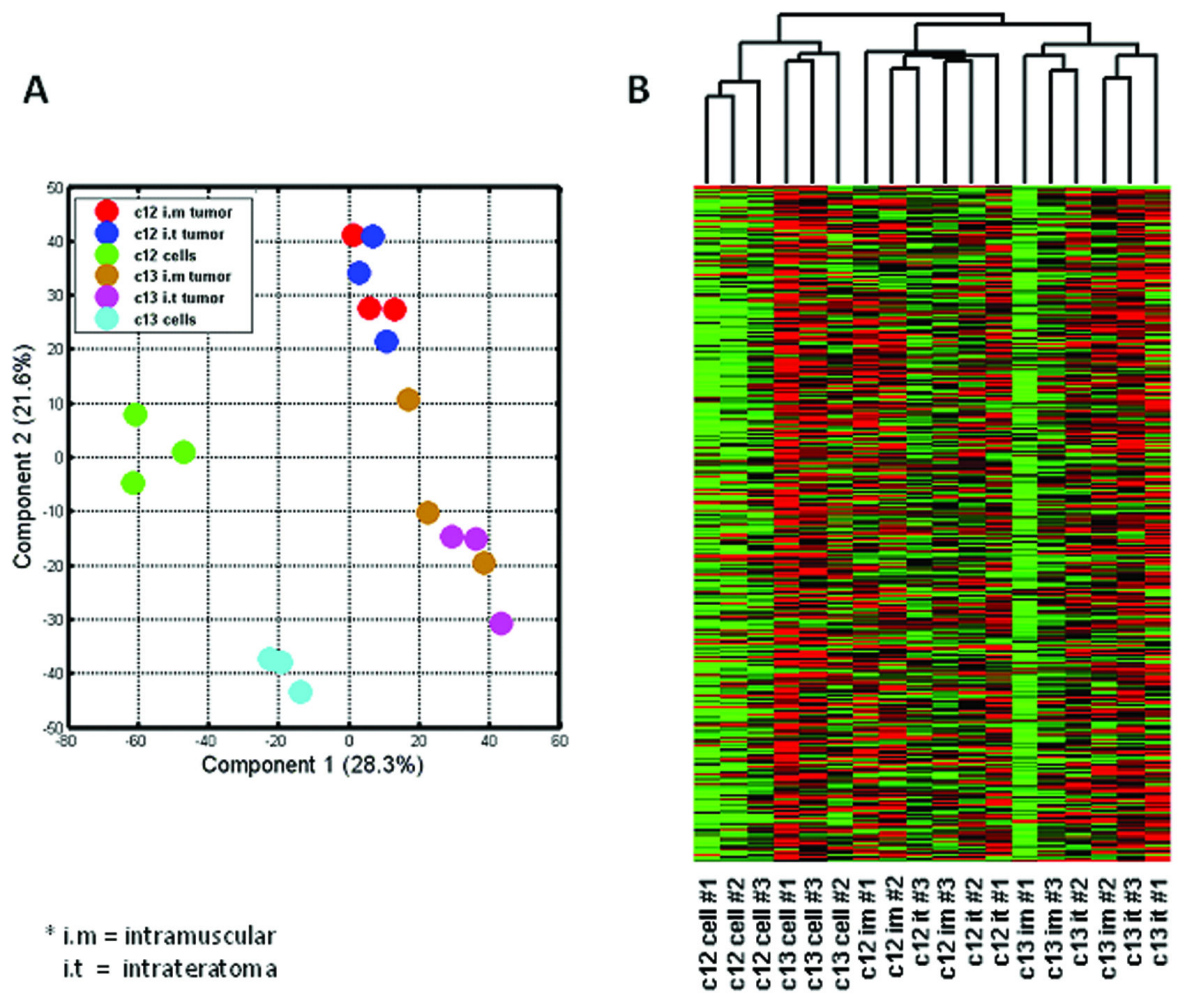
Principal component analysis (PCA) and hierarchical clustering of data sets. *A*, The PCA results are provided as two-dimensional representations based on contribution scores for the first two components. Discrimination between cancer cell subpopulations (CCSPs) C12 and C13 samples is shown as indicated in the color capture. *B*, Hierarchical clustering of the samples using all 48,803 probe elements on the Illumina bead chip demonstrated variability between CCSPs C12 and C13 samples.

We have recently reported the observation that consistent heterogeneity in tumorigenic properties among six different OCCC-derived CCSPs was most strikingly reflected in their niche-dependent *in vivo* tumorigenic capacities and tumor cellular phenotypes [36], and that the hESC–derived niche further supports self-renewing CSC and exposes their full repertoire of tumorigenic phenotypes [5]. In order to shed light on the tumor microenvironment contribution to this heterogeneity, we focused on gene expression microarray analyses to characterize the gene expression profiles of two distinct cancer cell subpopulations, CCSP C12 and C13 which grow successfully in both the xenotransplantation and the hESC-based teratoma models, and which exhibit the extremes of tumorigenic phenotypic attributes and niche-dependent self-renewal capacity [5,36]. C12-derived tumors are characterized by an abundance of highly differentiated ovarian structures, while C13-derived tumors exhibit poor ovarian structural differentiation [36]. In addition, C13 preserves its capacity for self-renewal as demonstrated by *in vivo* perpetuation of tumorigenic cancer cells both in the murine and the hESC-derived cellular tissue while C12 fails to perpetuate tumorigenic cells in the murine tissue, but generates highly aggressive and invasive tumors within the hESC-derived cellular tissue [5]. In the light of this striking effect, we aimed to delineate the gene expression profile of CCSP C12 and C13 derived tumors as a reflection of the interaction between the tumor cells and the tumor microenvironment. RNA samples were extracted from C12 and C13 grown in cell culture, and from tumors generated i.m and i.t (each sample in triplicate) and hybridized onto whole genome expression arrays (see Material and Methods). Schematic representation of the analysis procedure is described in Figure 1. The array analyses were performed at the following levels: C12 versus C13 *in vitro* grown cells, C12 versus C13 i.m tumors and C12 versus C13 i.t tumors (Figure 1A, B). Principal component analysis (PCA) for the data obtained was performed to assess the relative hierarchical contribution of differences in gene expression among the samples (Figure 2A). Distinct clustering of C12 *in vitro* grown cells samples and C13 *in vitro* grown cells samples was observed, however, an obvious relationship is demonstrated between C12 i.m and i.t samples and between C13 i.m and i.t samples, indicating significant differences in gene expression profiles between CCSPs C12 and C13. Hierarchical cluster analysis (HCA) of each sample in triplicates further confirms these results (Figure 2B). Differentially expressed genes (DEG) between C12 and C13 *in vitro* grown cells and between tumors generated i.m and i.t were identified based on fold change (>2) and corresponding statistical significant p-values (>0.05). Overlap of DEG across all the examined samples revealed 47 overlapping genes that were significantly changed (Figure 1A) which comprise the core differences between C12 and C13 cancer cell subpopulations, out of which, 26 and 21 genes demonstrated elevated expression in C12 and in C13 respectively (Tables 1 and 2). 

**Table 1:** Differentially expressed genes between cancer cell subpopulations (CCSPs) C12 and C13 *in* *vitro* grown cells and between tumors generated intramuscular (i.m) and intrateratoma (i.t).

**Elevated in CCSP C12**			**C12 vs C13 cells**		**C12 vs C13 i.m**		**C12 vs C13 i.t**	
**Illumina probe_ID**	**Gene Symbol**	**Gene Name**	**fold change**	**p-value**	**fold change**	**p-value**	**fold change**	**p-value**
ILMN_1677814	ABCC3	ATP-binding cassette, sub-family C (CFTR/MRP), member 3	10.03	0.002	12.40	0	10.40	0.003
ILMN_2081070	BTC	betacellulin	6.38	0.001	2.71	0	3.29	0
ILMN_1677108	CAPN13	calpain 13	5.80	0.012	11.07	0.001	5.93	0.005
ILMN_1777190	CFD	complement factor D (adipsin)	4.37	0.001	3.68	0	3.14	0.001
ILMN_1656560	DKFZP564O0823	prostate androgen-regulated mucin-like protein 1	5.25	0	3.78	0.001	3.83	0.004
ILMN_1815673	DKK3	dickkopf WNT signaling pathway inhibitor 3	2.63	0.025	6.38	0.003	6.27	0.004
ILMN_2398159	DKK3	dickkopf WNT signaling pathway inhibitor 3	3.21	0.009	8.70	0.001	8.49	0.001
ILMN_1729455	EML1	echinoderm microtubule associated protein like 1	3.15	0.010	5.68	0.003	5.68	0
ILMN_1743445	FAM107A	amily with sequence similarity 107, member A	29.68	0.009	17.21	0	16.30	0
ILMN_1715748	FLNC	filamin C, gamma	10.70	0	3.75	0.006	4.38	0.001
ILMN_1799744	GALC	galactosylceramidase	9.14	0.001	12.38	0.001	13.48	0
ILMN_1726666	GPX3	glutathione peroxidase 3 (plasma)	169.14	0	409.81	0	368.95	0
ILMN_1696183	HBQ1	hemoglobin, theta 1	7.68	0	2.99	0	3.02	0
ILMN_2217329	IAH1	isoamyl acetate-hydrolyzing esterase 1 homolog (S. cerevisiae)	13.43	0	9.45	0	6.93	0
ILMN_1673521	KISS1R	KISS1 receptor	5.62	0	4.14	0	3.97	0
ILMN_1662358	MX1	myxovirus (influenza virus) resistance 1, interferon-inducible protein p78 (mouse)	17.36	0	39.97	0.001	27.66	0
ILMN_1709814	NMRAL1	NmrA-like family domain containing 1	8.74	0	9.41	0	10.10	0
ILMN_1680339	PDGFRL	platelet-derived growth factor receptor-like	2.95	0.003	5.20	0.001	5.31	0.001
ILMN_1792506	PLA1A	phospholipase A1 member A	4.84	0	2.49	0	2.84	0.003
ILMN_1736533	RND2	Rho family GTPase 2	6.80	0.001	7.36	0.001	6.50	0.002
ILMN_1726928	TCEA3	transcription elongation factor A (SII), 3	12.49	0	3.97	0.001	5.62	0
ILMN_1760245	TMEM42	transmembrane protein 42	6.78	0.001	5.26	0.001	5.19	0
ILMN_1713990	TRIP6	thyroid hormone receptor interactor 6	3.37	0.001	6.21	0.001	5.12	0.004
ILMN_1670377	ZNF20	zinc finger protein 20	3.95	0.001	6.49	0	5.68	0
ILMN_1798533	ZNF22	zinc finger protein 22	7.72	0	6.72	0	6.57	0
ILMN_2117904	ZNF22	zinc finger protein 22	7.69	0	6.51	0	7.66	0
ILMN_1728710	ZNF816A	zinc finger protein 816	6.51	0.001	7.04	0.001	7.22	0
ILMN_1802053	ZNF91	zinc finger protein 91	4.18	0.006	3.90	0	3.77	0.001

Values of zero (0) = less than 0.001

**Table 2:** Differentially expressed genes between cancer cell subpopulations (CCSPs) C13 and C12 *in* *vitro* grown cells and between tumors generated intramuscular (i.m) and intrateratoma (i.t).

**Elevated in CCSP C13**			**C13 vs C12 cells**		**C13 vs C12 i.m**		**C13 vs C12 i.t**	
**NAME**	**Gene Symbol**	**Gene Name**	**fold change**	**p-value**	**fold change**	**p-value**	**fold change**	**p-value**
ILMN_1802167	ALDH1L1	aldehyde dehydrogenase 1 family, member L1	5.11	0	3.87	0.001	6.57	0.001
ILMN_1805410	C15orf48	chromosome 15 open reading frame 48	8.38	0.007	3.06	0.009	2.83	0.002
ILMN_1774287	CFB	complement factor B	4.45	0.001	3.50	0.001	5.35	0
ILMN_1810942	CYP3A5	cytochrome P450, family 3, subfamily A, polypeptide 5	4.87	0.011	5.76	0.017	9.23	0.007
ILMN_1725597	FXYD4	FXYD domain containing ion transport regulator 4	4.57	0.005	6.08	0.005	6.80	0.004
ILMN_1660973	GAD1	glutamate decarboxylase 1	5.44	0.001	2.52	0.002	2.55	0.001
ILMN_1712082	GCNT3	glucosaminyl (N-acetyl) transferase 3, mucin type	5.90	0.009	3.65	0	4.19	0.002
ILMN_1739813	HYAL1	hyaluronoglucosaminidase 1	4.03	0.008	15.84	0	14.14	0
ILMN_2314417	HYAL1	hyaluronoglucosaminidase 1	2.43	0.004	6.05	0.002	5.65	0.001
ILMN_1778010	IL32	interleukin 32	2.87	0.009	6.45	0.001	5.18	0
ILMN_1705814	KRT80	keratin 80	3.99	0.015	3.84	0.002	3.57	0
ILMN_1692223	LCN2	lipocalin 2	20.32	0.005	2.50	0.002	3.82	0.006
ILMN_1814270	LY6D	lymphocyte antigen 6 complex, locus D	10.29	0	9.52	0.002	12.99	0
ILMN_1802780	M160	CD163 molecule-like 1	3.29	0.001	5.32	0.004	4.83	0.001
ILMN_1651568	MUC13	mucin 13, cell surface associated	4.57	0.005	3.98	0.002	7.13	0
ILMN_1709809	NHP2L1	NHP2 non-histone chromosome protein 2-like 1 (S. cerevisiae)	11.14	0	7.95	0	5.26	0.001
ILMN_1773389	PLTP	phospholipid transfer protein	6.38	0	3.28	0.003	4.61	0.006
ILMN_1713829	PTGES	prostaglandin E synthase	5.91	0	8.53	0	9.18	0
ILMN_1651429	SELM	selenoprotein M	2.53	0.001	3.51	0.002	3.80	0.001
ILMN_1683670	SLC10A2	solute carrier family 10 (sodium/bile acid cotransporter), member 2	5.65	0.001	15.11	0	5.14	0.006
ILMN_1739001	TACSTD2	tumor-associated calcium signal transducer 2	76.15	0	5.31	0.012	5.46	0.004
ILMN_1725387	TMEM200A	transmembrane protein 200A	4.54	0.004	7.31	0.004	7.95	0.003

Values of zero (0) = less than 0.001

### Microenvironment - dependent tumor gene expression profile

**Figure 3:**
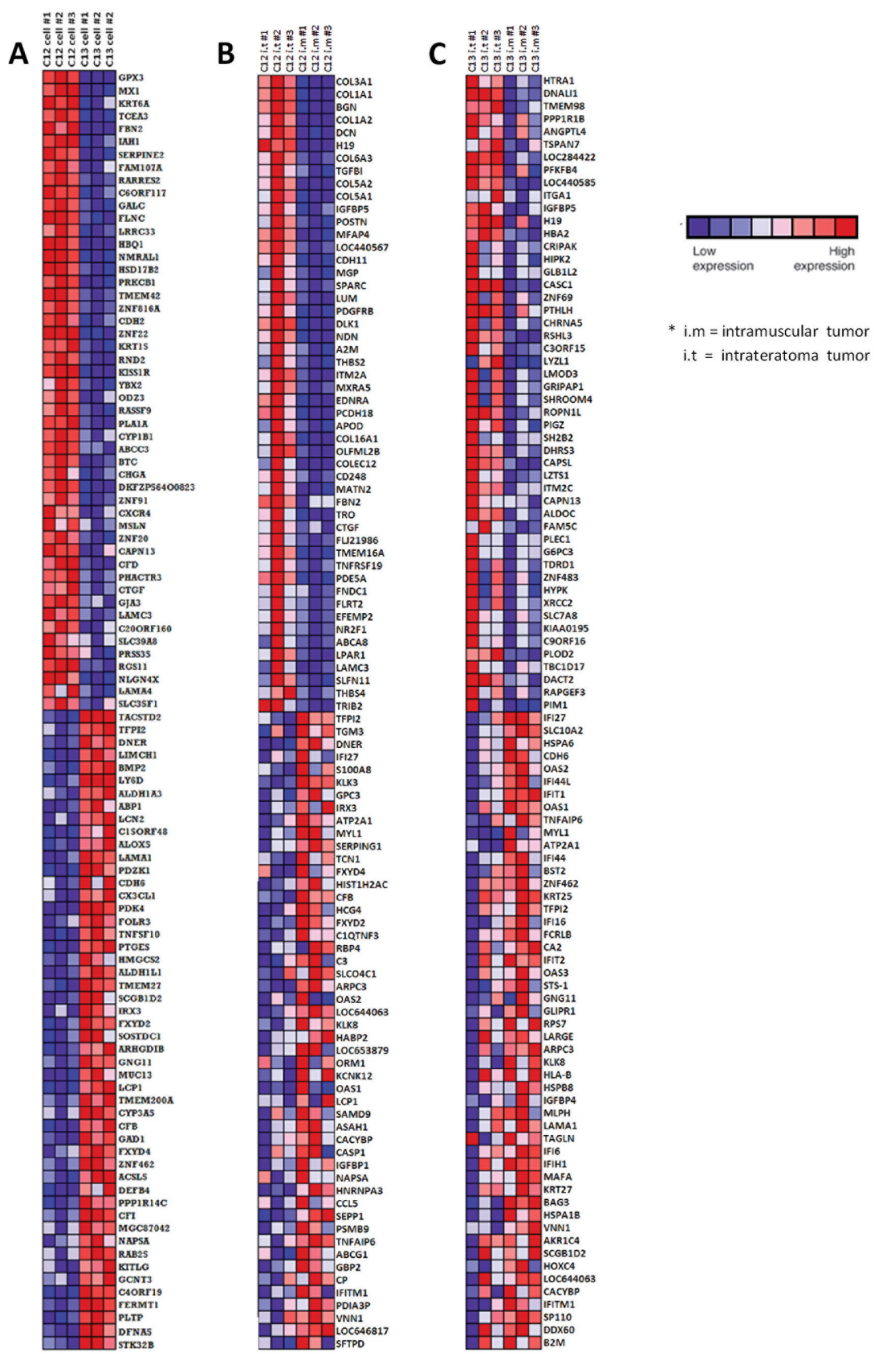
Heat Map of differentially expressed genes ranked by Gene Set Enrichment Analysis (GSEA). The 100 most differentially expressed genes between cancer cell subpopulations (CCSPs) C12 and C13 *in* *vitro* grown cells (*A*), CCSP C12-derived tumors generated intramuscular (i.m) and intrateratoma (i.t) (*B*) and CCSP C13-derived tumors generated i.m and i.t (*C*). The differential expression of genes was calculated according to the Signal-To-Noise metrics. The top 50 symbols represent genes that were elevated in tumors developed i.t. The next 50 symbols represent genes that elevated in tumors developed i.m. Genes which are located higher in each of the two 50 gene groups indicates a greater difference level than genes located at lower positions. Expression values are represented as shown in the color caption.

To examine the gene expression profile of CCSP C12 and C13 – derived tumors as a reflection of the interaction between the tumor cells and the tumor microenvironment (Figure 1B), we applied gene set enrichment analysis (GSEA) [39] to assess whether distinct gene sets were statistically significant. For this purpose we used a total of 3279 gene sets which are published in the Molecular Signatures Database of GSEA official website (http://broadinstitute.org/gsea). GSEA detects enrichment of whole sets of functionally-related groups of genes by comparing expression data with selected gene sets from available gene set databases. The gene sets with nominal p-value <0.05 (NOM p) and FDR q-value <0.05 were used to minimize identification of false positives in the GSEA analysis. Of the 3279 gene sets which were tested in the C12 i.m versus i.t comparison, 401 gene sets were enriched in C12 i.t, and 43 gene sets enriched in C12 i.m. Moreover, in the C13 i.m versus i.t comparison, 120 sets were enriched in C13 i.t class, and 43 gene sets were correlated with the C13 i.m class (Table 3). In order to interpret this large amount of data effectively, we used the LEA tool of the GSEA software, extracting the core gene members of the enriched gene sets that contribute the most to the corresponding ES values. By taking into consideration only leading edge genes, we generated 4 gene lists representing the most significant subsets for C12 i.m, C12 i.t, C13 i.m and C13 i.t. Figure 3 shows the 100 most differentially expressed genes ranked by GSEA for C12 and C13 *in vitro* grown cells (Figure 3A) and for C12 i.m and i.t (Figure 3B) and C13 i.m and i.t. (Figure 3C). 

**Table 3:** Representative enriched gene sets for CCSPs C12 and C13 –derived tumors generated intramuscular (i.m) and intrateratoma (i.t).

**Enriched gene sets**						
	**Gene Set Name Ver.2.5**	**Gene Set Size**	**ES**	**NES**	**NOM p**	**FDR q**
**C12 i.m**	DAC_IFN_BLADDER_UP	17	-0.755	-2.277	0	0
	GNF2_IGF1	25	-0.667	-2.182	0	0.002
	GNF2_LYN	26	-0.630	-2.150	0	0.003
	MODULE_345	114	-0.495	-2.223	0	0.003
	MODULE_436	124	-0.449	-2.082	0	0.006
	MODULE_71	21	-0.633	-2.014	0	0.01
	IFN_BETA_UP	65	-0.477	-1.967	0	0.011
	MODULE_171	128	-0.428	-1.979	0	0.014
	PROTEASOME	17	-0.588	-1.762	0.006	0.045
	ANDROGEN_AND_ESTROGEN_METABOLISM	23	-0.552	-1.791	0.01	0.038
**C12 i.t**	HSA04512_ECM_RECEPTOR_INTERACTION	85	0.794	2.652	0	0
	HSA01430_CELL_COMMUNICATION	135	0.708	2.516	0	0
	DNA_REPLICATION_REACTOME	44	0.691	2.104	0	0.002
	CANCER_UNDIFFERENTIATED_META_UP	68	0.641	2.083	0	0.003
	HSA04350_TGF_BETA_SIGNALING_PATHWAY	88	0.572	1.945	0	0.012
	CELL_ADHESION	171	0.518	1.867	0	0.022
	HSA04310_WNT_SIGNALING_PATHWAY	147	0.515	1.827	0	0.03
	CELL_CYCLE	77	0.565	1.852	0.001	0.024
	HSA05217_BASAL_CELL_CARCINOMA	55	0.617	1.940	0.001	0.013
	SRC_ONCOGENIC_SIGNATURE	58	0.562	1.753	0.006	0.044
**C13 i.m**	BECKER_TAMOXIFEN_RESISTANT_UP	38	-0.487	-3.126	0	0
	DSRNA_UP	36	-0.436	-2.132	0	0
	CMV_8HRS_UP	32	-0.451	-2.088	0	0
	MODULE_355	28	-0.416	-2.109	0	0
	DAC_IFN_BLADDER_UP	17	-0.535	-2.213	0	0.001
	HPV31_DN	48	-0.379	-2.167	0	0.002
	CMV_24HRS_UP	72	-0.263	-1.261	0	0.007
	NF90_UP	24	-0.487	-1.926	0	0.008
	RIBAVIRIN_RSV_UP	22	-0.409	-1.798	0	0.016
	ET743_RESIST_UP	17	-0.385	-1.689	0	0.036
**C13 i.t**	HSA01032_GLYCAN_STRUCTURES_DEGRADATION	30	0.593	1.590	0	0.002
	MYC_ONCOGENIC_SIGNATURE	190	0.494	1.521	0	0.006
	RAS_ONCOGENIC_SIGNATURE	249	0.449	1.396	0	0.029
	HSA04910_INSULIN_SIGNALING_PATHWAY	135	0.454	1.386	0.001	0.032
	HSA04012_ERBB_SIGNALING_PATHWAY	87	0.485	1.433	0.002	0.019
	HSA04370_VEGF_SIGNALING_PATHWAY	70	0.482	1.415	0.004	0.023
	HSA04150_MTOR_SIGNALING_PATHWAY	48	0.520	1.477	0.005	0.011
	HSA04512_ECM_RECEPTOR_INTERACTION	85	0.454	1.335	0.012	0.052
	HSA04330_NOTCH_SIGNALING_PATHWAY	46	0.488	1.381	0.02	0.033
	HSA00100_BIOSYNTHESIS_OF_STEROIDS	24	0.542	1.418	0.031	0.022

Values of zero (0) = less than 0.001

MSigDB Ver.2.5

ES = Enrichment Score NES = Normalized Enrichment Score FDR = False Discovery Rate

### Gene Ontology (GO) enrichment analysis

**Figure 4:**
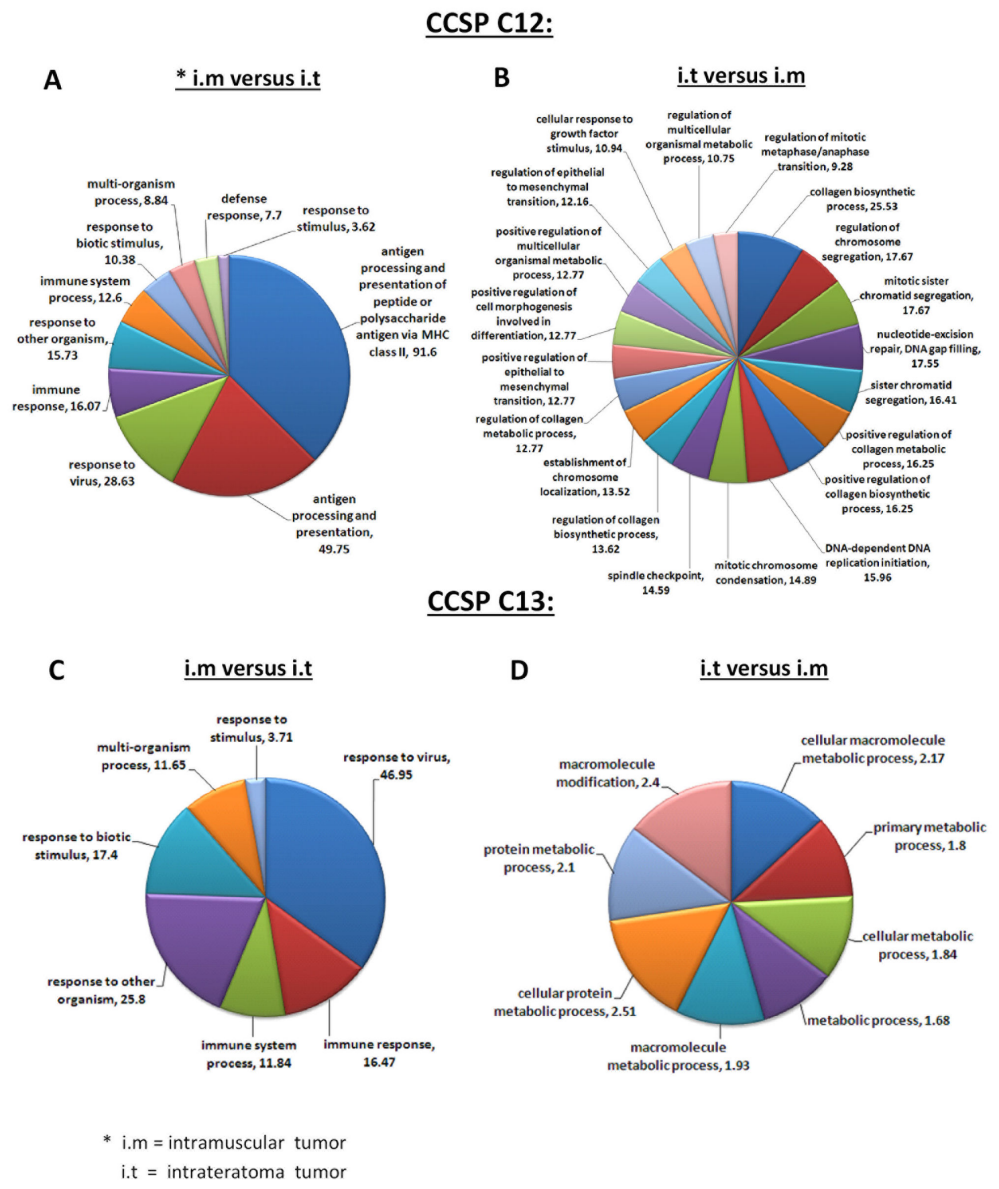
Gene Ontology (GO) annotations for cancer cell subpopulations (CCSPs) C12 and C13-derived tumors generated intramuscular (i.m) and intrateratoma (i.t). Statistically significant GSEA gene sets were subjected to leading edge analysis (LEA) and the resulting genes were grouped in ontological annotations of biological process categories. The pie charts present the enriched GO annotations and their matching enrichment scores (ES) for: *A*, GO annotations upregulated in C12 tumors generated i.m compared with C12 tumors generated i.t. *B*, GO annotations upregulated in C12 tumors generated i.t compared with C12 tumors generated i.m (the 20 GO annotations with the highest enrichment scores are shown). *C*, GO annotations up regulated in C13 tumors generated i.m compared with C13 tumors generated i.t. *D*, GO annotations upregulated in C13 tumors generated i.t compared with C13 tumors generated i.m.

The resulting gene lists were subjected to GO enrichment analysis [40], using the GOrilla web based software. The input for this analysis consisted of lists of leading edge genes and a background list which represents all the genes tested in the Illumina gene expression platform. With a conservative criterion of Bonferroni adjusted p-value < 0.005, we obtained two lists of enriched GO terms per CCSP, representing its interaction with the two different microenvironments. Importantly, in the murine niche, only a limited number of differences between the two CCSPs were found (Figure 4A, C). 10 and 7 GO terms were enriched in CCSP C12 and C13 respectively. All the 7 GO terms that were enriched in CCSP C13 i.m were also enriched in CCSP C12 i.m with similar enrichment scores (ES) values. All such designated GO terms are related to the immune response system and involved in the “cell response to a stimulus produced by a living organism”. The enrichment of these GO terms demonstrates a change in the state or activity of the CCPSs as a result of interaction with the heterologous external environment as would be expected. On the other hand, in the hESC-derived microenvironment far greater differences between the two CCSPs were observed (Figure 4B, D). CCSP C12 exhibited enrichment of multiple GO terms related to cell cycle, which might reflect the effect of the hESC-derived microenvironment as a more supporting niche in contrast to the murine microenvironment. Moreover, GO terms related to the process of epithelial to mesenchymal transition (EMT) were also enriched in C12 derived tumors in the hESC-derived microenvironment. Relative to C12, CCSP C13 derived tumors in the hESC-derived microenvironment exhibit only a few, low ES value enriched GO terms, mostly related to metabolic processes. The lists of enriched GO terms in C12 and C13 – derived tumors generated i.m and i.t are presented in Table S1. 

Taken together, this analysis demonstrates the different pathways of interactions between CCSPs C12 and C13 with the microenvironment during the process of tumor progression. In contrast to the conventional murine model, the hESC-based model demonstrates a more complex cancer cell-microenvironment interaction, which may provide a more authentic reflection of the correspondent relationship between the tumor cells of a patient and the complex human tissues surrounding the tumor.

### The role of Wnt, Notch and Hedgehog (Hh) signaling pathways in the niche-dependent maintenance of the self-renewal capacity of CCSPs C12 and C13

The gene expression array analyses in our multiple independent studies were conducted under rigorous statistical constraints to ensure that significant differences would be valid and of biological significance (see Materials and Methods). The results of this analysis highlighted several pathways that are involved in cell cycle regulation, regulation of cell-cell adhesion, and antigen processing and presentation, with clear-cut differences in expression levels among tumors generated using the two *in vivo* models. Representative enriched gene sets for C12 and C13 – derived tumors generated im and i.t indicated among others, evidence for the role of Wnt and Notch signaling pathways in the niche-dependent maintenance of the self-renewal capacity of C12 and C13 (Table 3). The Wnt Notch and Hh signaling pathways have been demonstrated to be involved in directing growth and patterning during embryonic development, as well as to be responsible for the control of self-renewal and differentiation in various stem cells and CSC [41-43]. 

**Figure 5:**
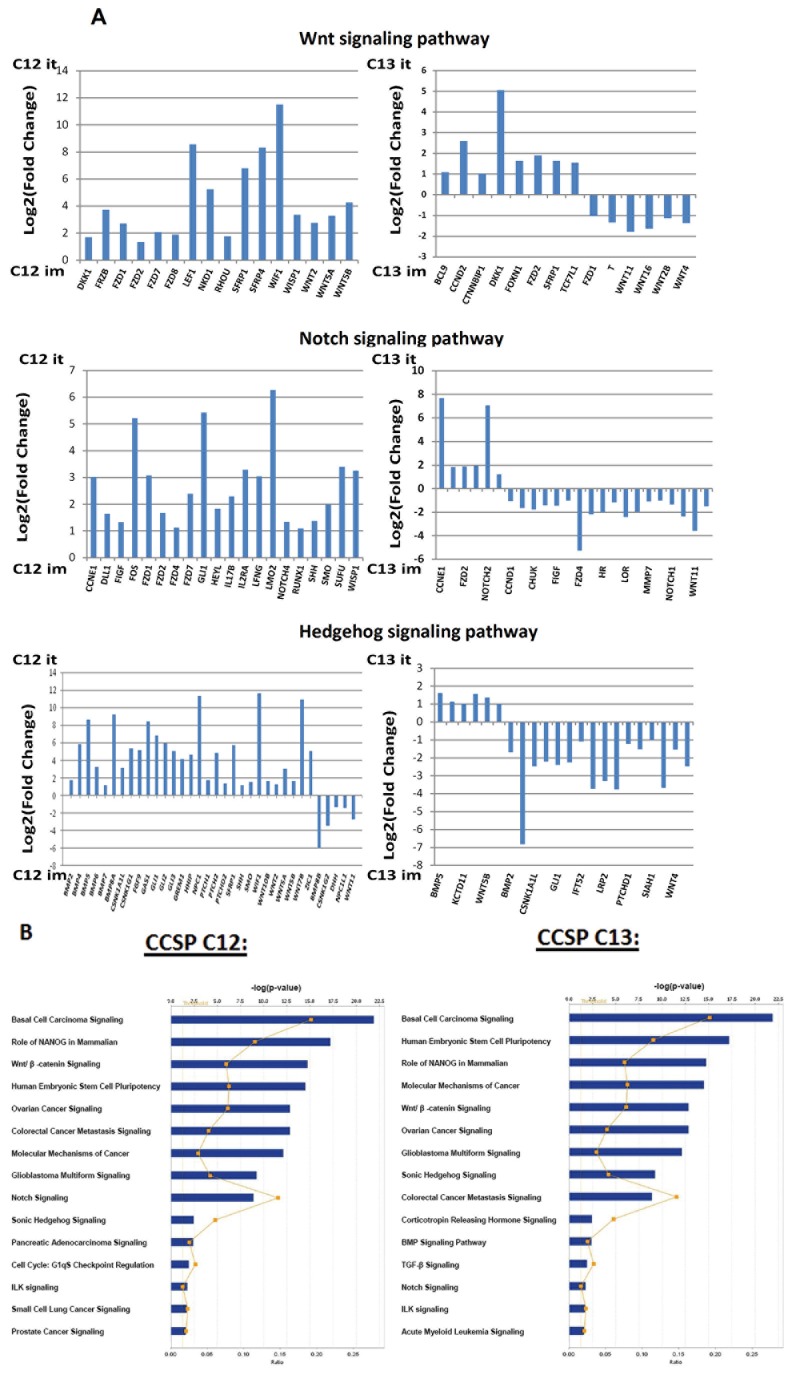
WNT, Notch, and Hedgehog signaling pathway arrays analyses. A comparison analysis of gene expression was performed using the RT^2^ Profiler PCR SuperArrays technique. *A*, Using a threshold value of 2-fold expression change and a statistical significance of p-value < 0.05, 69 and 54 differentially expressed genes in these 3 pathways were identified between CCSPs C12 and C13 tumors generated intramuscular (i.m) and intrateratoma (i.t) as indicated. *B*, Ingenuity Pathways Analysis (IPA) demonstrates 10 statistically significant biological functions with the highest significance score common but ranked differently by p-values in C12 and C13 tumors generated i.t as indicated. The upper X-axis is the reciprocal of the p-values and the lower X-axis and orange squares indicate the ratio between altered genes and the total number of genes in the particular pathway. The threshold line marks p-value = 0.05.

To further evaluate the contribution of Wnt, Notch and Hh signaling pathways to the niche-dependent maintenance of the self-renewal capacity of CCSPs C12 and C13 in tumors generated within the hESC-based model, we performed a comparison analysis of gene expression by using the RT^2^ Profiler PCR SuperArrays technique. The multigene profiling capabilities of such a technique provide quantification of both rare and abundant genes in the same sample and might address the diverse results obtained from gene expression microarray analyses due to utilization of various normalization methods [44]. Using a threshold value of 2-fold expression change, 69 and 54 differentially expressed genes in these 3 pathways were identified between C12 and C13 tumors generated i.m and i.t (respectively) (Figure 5A). 

Expression of several Wnt Notch and Hh signaling pathways negative and positive regulators were increased when comparing C12 i.t versus i.m tumor and C13 i.t versus i.m tumor (Figure 5A and Figures S1, S2 and S3). To investigate possible biological interactions of Wnt, Notch and Hh signaling pathways - related differentially regulated genes, datasets representing genes with an altered expression profile between C12 and C13 i.m and i.t tumors were imported into the Ingenuity Pathway Analysis Tool. The list of differentially expressed genes analyzed by IPA revealed 15 statistically significant biological pathways, ten of which are common to C12 and C13 tumors (Figure 5B). Of these top canonical pathways, three common categories were observed: 1. Cancer related signaling, including basal cell carcinoma signaling, ovarian cancer signaling, colorectal cancer metastasis signaling, molecular mechanism of cancer and glioblastoma multiforme signaling. 2. Stem cell related functions including the NANOG pathway for mammalian embryonic stem cell pluripotency, human embryonic stem cell pluripotency, WNT/β-catenin signaling, Sonic Hh signaling and Notch signaling. 3. Integrin-linked kinase ILK signaling which has been associated with cell migration, proliferation, adhesion, and signal transduction. A change in the cell cycle G1/S checkpoint regulation was identified between C13 i.m and i.t tumors, which might support the role of the hESC-derived cellular microenvironment in self-renewal mechanisms.

Taken together, the RT-PCR array profiling of Wnt, Notch and Hh pathways might indicate the involvement of these pathways in tumor complexity in a microenvironment-dependent manner. 

### Corroboration and analysis of highly significant array targets

**Figure 6:**
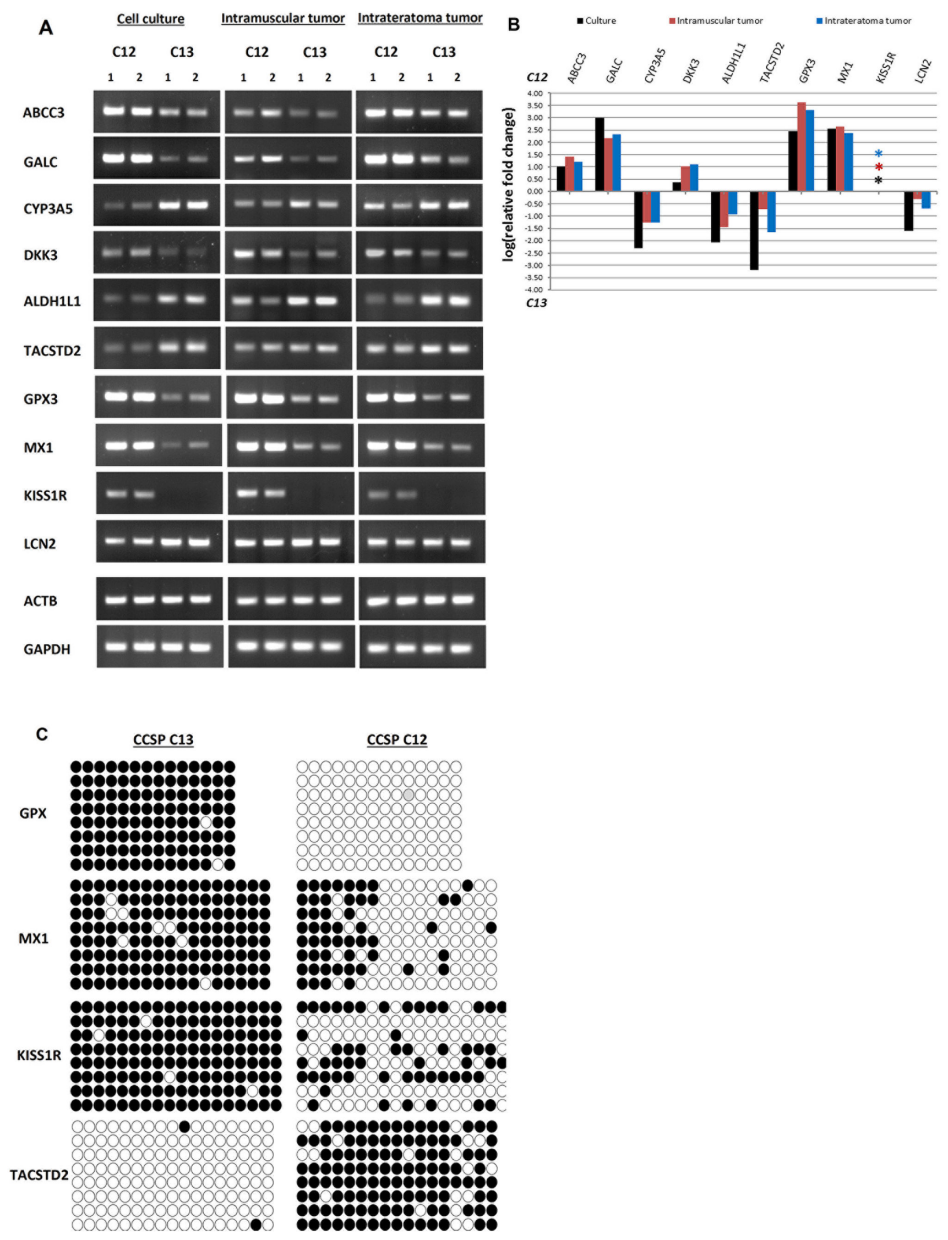
Validation of the gene expression microarray data. Total RNA extracted from CCSPs C12 and C13 *in* *vitro* grown cells and from C12 and C13 – derived tumors generated intramuscular (i.m) and intarteratoma (i.t) were analyzed by quantitative real-time RT-PCR using specific primers as indicated (each in 2 independent RNA samples). *A*, DNA products were separated on 2% agarose gel and *B*, the bars demonstrate the relative fold change in expression levels of 10 differentially expressed genes. ACTB and GAPDH were used for internal controls. Asterisk indicates that no KISS1R expression was observed in the C13 samples. These experiments were performed twice, each sample in quadruplicates. *C*, Bisulfit sequencing analysis of GPX3, MX1, TACSTD2, and KISS1R promoter regions in CCSPs C12 and C13. Open circles represent unmethylated CpG dinucleotides and closed circles represent methylated CpG dinucleotides. Each row is derived from an individual subclone. These experiments were performed twice, each sample in quadruplicates.

In order to verify that the differential gene expression values observed are not the result of inherent limitations of the array technology, we have validated the gene expression values by quantitative real time RT-PCR using SYBR Green I dye detection with product verification using melting curve analysis. As demonstrated in Figure 1 and in Tables 1 and 2, 26 genes which were comparatively elevated in CCSP C12 and 21 genes which were comparatively elevated in CCSP C13 were categorized into four groups of interest. Accordingly, we chose 1-2 genes from each group for validation analysis as follows: 1) Drug resistance related genes: ABCC3 product is involved in multi-drug resistance which provides protection from chemotherapeutic agents in various cancer cell subpopulations [45], GALC encodes the glucosylceramide enzyme, which is involved in mechanism of drug resistance in cancer cells [46] and CYP3A5 which encodes for a protein that catalyzes reactions involved in drug metabolism [47]. 2) Stem cell associated genes: DKK3 encodes for a protein that acts as an inhibitor of the Wnt pathway, which is known for its role in directing growth and patterning during embryonic development and is responsible for the control of self-renewal and differentiation in various stem cells and CSC [48]. ALDH1L1 is a member of the ALDH1 family whose increased activity has been implicated with cells having stem/progenitor properties [49]. TACSTD2/TROP2, a cell surface glycoprotein, is a marker of human prostate basal cells with stem cell characteristics and a putative role in signal transduction [50]. 3) Tumorigenic process related genes: GPX3 encodes for an enzyme which plays a critical role in detoxifying reactive oxidative species, was found to be widely inactivated in prostate cancers [51]. GPX3 has also been suggested as a novel tumor suppressor gene due to its ability to reduce prostate cancer cell invasiveness, tumor volume and metastasis. KISS1R is a human metastasis suppressor gene whose product, metastin, has a potential role in modulating the biologic behavior of papillary carcinomas [52]. MX1 gene product promotes cell death induced by apoptotic stimuli and therefore low levels of MX1 protein might contribute to apoptosis resistance during cancer development [53]. 4) Cell morphology related genes: The LCN2 gene encodes a secretory glycoprotein, which plays a role in epithelial to mesenchymal transition (EMT) and elevates cell motility and invasiveness [54]. The results of the validation analysis presented in Figure 6 A, B, confirm the differences in expression levels of various genes as detected by utilizing the gene expression array analysis.

### Intratumoral heterogeneity of epigenetic regulation of gene expression

The list of highly significant array targets which were validated and analyzed using qRT-PCR, included four genes which have been previously reported to be epigenetically regulated in various types of cancers [55,56]. The GPX3, MX1 and KISS1R genes were categorized into the group of tumorigenic process related genes and demonstrated elevated expression levels in CCSP C12 compared with C13 in *in vitro* grown cells, i.m and i.t tumors as follows: GPX3: 279, 4066, and 2067 fold induction respectively; MX1: 350, 431 and 235 fold induction and KISS1R which demonstrated high levels of expression in C12 but was not expressed in C13. On the other hand, TACSTD2/TROP2 gene was categorized into the group of stem cell associated genes and demonstrated a reduced expression level in C12 compared with C13: 1562, 5.3 and 46 fold respectively (Figure 6 A, B). Since transcriptional silencing associated with aberrant promoter hypermethylation is a common mechanism of gene inactivation in cancer cells, we have examined whether the DNA methylation status of these genes' promoters correlates with diminished gene expression in the CCSPs. For this purpose, genomic DNA of each of the specific genes was bisulfite converted, amplified, cloned, and sequenced. A high frequency of methylated CpG sites was detected on the promoter region of GPX3, MX1, and KISS1R genes in CCSP C13 cells, whereas in CCSP C12, very few CpG sites were methylated (Figure 6C). These results correlate with reduced transcription level of these genes in CCSP C13. On the other hand, a high frequency of methylated CpG sites were observed in the promoter region of the TACSTD2/TROP2 gene in CCSP C12 which correlates with reduced TACSTD2/TROP2 expression level in CCSP C12 in comparison to CCSP C13 (Figure 6C). Of note, TACSTD2/TROP2 gene is transcriptionally active in CCSP C13 which exhibits stable and durable *in vivo* self-renewal and tumorigenic differentiation capacity as opposed to CCSP C12 [5]. 

Our results confirm previous publications demonstrating epigenetically based decreased transcription levels of these genes as a result of gene promoter hypermethylation [57,58], and extend this to the level of intratumoral heterogeneity of cancer cells.

## Discussion

The clinical application of the CSC paradigm requires extensive research efforts for developing combined anti-cancer therapeutic strategies to suppress CSC with self-renewal capacity in addition to non-CSC that together comprise a distinct tumor in order to prevent recurrence. Moreover, such therapeutic strategies must take into consideration the variability of CSC frequencies in various solid tumors and the heterogeneity in the self-renewing populations of CSC within an individual tumor [59]. The development of such therapies will also require adequate preclinical *in vivo* experimental systems which will reflect the whole spectrum of intrinsic and environmental properties of various cancer cells comprising the tumor, to determine the mechanisms underlying its growth and progression. Since the balance between self-renewing CSC and non-self-renewing differentiated cancer cells depends on cell-cell and cell-microenvironment interactions, the conventional xenograft of human cancer cells within immunodeficient mice might not always reflect the repertoire of target cancer cell subpopulations within a given patient tumor [60]. The novel experimental model we have established in which human cancer cells grow within a hESC-derived cellular microenvironment provides growth support for a much wider spectrum of CSC populations from an individual tumor [29]. 

The crucial contribution of *in vivo* models in defining self-renewal and differentiation properties of cancer cells is also expressed in the potential to recapitulate of the entire heterogeneous entity of a tumor. This feature is especially amenable to investigation in OCCC with its striking intratumoral heterogeneity that might also account for resistance to anti-cancer therapies [31,35,61]. We have demonstrated that the tumorigenic potential of OCCC – derived heterogeneous cancer cell subpopulations depends on the niche and on the capacity of the cancer cells to recruit stromal cells into the tumor tissue [5,36]. Although global gene expression profiling distinguished OCCC from other poor-prognosis ovarian carcinomas as reflected by histological phenotype and biological behavior [62,63], intratumoral heterogeneous gene expression profiles of distinct cancer cell subpopulations were not examined in previous studies. 

The two CSC populations - CCSPs C12 and C13 - used for the current study were chosen out of multiple distinct CSC populations present in an individual OCCC tumor based on their histological and immunohistochemical phenotypes and on the basis of their functional properties using the *in vivo* transplantation propagation assay [36]. The highly differentiated C12 tumor type, and the C13 poorly differentiated tumor, demonstrate also a stable surface phenotype of CD44^+^/CD24^+^ and ALDH activity, which has been related to self-renewing CSC. C12 and C13 derived tumors generated within the hESC-derived cellular microenvironment, indicate a shift of the balance from a combination of self-renewal and tumorigenic differentiation towards a predominant self-renewal phenotype. Thus, we postulate that the hESC-based *in vivo* experimental model would be more suitable for understanding the tumor multiple phenotypes and molecular underpinnings including its interactions with the microenvironment and will enable the development of appropriate therapeutic strategies for suppression of key subpopulations of self-renewing CSCs for tumor control [36]. 

First and foremost, the gene expression microarray analyses, clarify gene expression and epigenetic signatures underlying the influence of the niche on the balance between self-renewing and non-self-renewing cancer cells in tumors generated by heterogeneous CSC populations derived from an individual tumor. 

The approach we have used included generating CCSP C12 and C13 – derived tumors in the murine tissue and in the hESC-derived microenvironment, preparation of frozen sections from each tumor, laser microdissection and pressure catapulting (LMPC) the tumor tissue samples, extraction of RNA and then conducting expression array analyses. Such a strategy allowed the examination of the gene expression profile of the tumors as a reflection of the interaction between the various tumor cells and the tumor microenvironment. The analyses were performed at several levels which include comparisons of C12 and C13 *in vitro* grown cells, i.m and i.t tumors and indeed, PCA and HCA exhibited different expression profiles for C12 and C13 *in vitro* and *in vivo* confirming the existence of distinct CSC populations in the tumor. In order to detect the core list of genes which differ between the various groups, a very stringent analysis approach was taken although we were aware of the fact that certain genes will not be detected, since the aim of this strategy was to obtain a global characterization of the interactive pathways between intratumoral heterogeneous cancer cells. Within the list of 47 genes which represent the core differences between C12 and C13 expression profile, certain genes such as TACSTD2/TROP2 and LNC2 demonstrated higher or lower (respectively) fold change expression level in *in vitro* grown cells in contrast to C12 and C13 tumors (Tables 1 and 2), indicating the influence of the microenvironment on the expression of these genes. The GSEA approach also demonstrated a different predictive gene signature for C12 and C13 subpopulations for the two different *in vivo* models. These findings are further underscored by the enriched GO terms identified for C12 and C13 tumors generated i.m and i.t. The ontological annotations of biological processes categories identified for C13 – derived tumors generated i.m included mainly GO terms related to the immune response as an outcome of the *in vivo* xenograft model. For C13 – derived tumors generated i.t mainly GO terms related to metabolic processes were identified. On the other hand, although C12 i.m tumors exhibit GO terms similar to C13 i.m tumors, the C12 i.t tumors exhibit enriched GO annotations categories, indicating the massive interaction of C12 cancer cells with the surrounding stroma. These results might explain the extensive recruitment of stromal cells into C12-derived tumors and in particular, the ability of C12 tumors to perpetuate within the hESC-derived cellular tissue as opposed to the murine tissue [36]. These results also point at the differences between C12 and C13 CSC populations in that C13 cells might present stable and aggressive CSC population with niche- independent intrinsic capacity for self-renewing and tumorigenic differentiation, while the CSC properties of C12 cell population are evident in a niche –dependent manner. Furthermore, we postulate that distinct CSC populations present different requirements from the microenvironment to accomplish their tumorigenic potential, hence, the microenvironment attributes actually determine the tumorigenic capacity of the specific CSCs. Of note, the fact that C13 tumors perpetuated in recipient mice demonstrate an alteration of their histological phenotype from poorly differentiated to a highly differentiated type of tumor similar to C12 tumor, might indicate a reduction of C13 intrinsic capacities and elevation of its dependency on the surrounding stroma. These observations suggest that changing the balance from self-renewal into tumorigenic differentiation processes might enhance the vulnerability to anti-cancer therapies. 

The qRT-PCR based validation confirmed the results obtained in the microarray analysis and point at various aspects of intratumoral cancer cell heterogeneity by demonstrating differential expression of genes related to the cell morphology, stemness properties, and specifically drug resistance. 

Taken together, the current results using OCCC provide clear evidence for the gene expression and epigenetic determinants of intratumoral heterogeneity of cancer cells in a model system with correspondingly well-characterized clinical relevant phenotypic heterogeneity. The RT-PCR array profiling of Wnt, Notch and Hh signaling pathways importantly includes differences between the two *in vivo* models in key self-renewal pathways involved in inducing cancer cell proliferation and self-renewal processes, highlighting the larger repertoire of biological processes involved in supporting tumor growth within the hESC-based tissue. Further studies in this direction will be needed to demonstrate the role of these signaling pathways in the biology of the subpopulations of cancer cells. Although epigenetic – based silencing of gene expression has been demonstrated in a wide variety of tumor cells, this study presents clear evidence for differential intratumoral epigenetic changes. Extension of the findings using the hESC-derived microenvironment to other tumors will be needed to demonstrate broad relevance. To date, reproducibility of our previously published results has been demonstrated in two independent publications indicating that the hESC-based model express *bona fide* human tumor blood vessels and enhance tumor engraftment rate by primary human ovarian CSC-like [37], and that the hESC-based model enable the implantation and growth of childhood neuroectodermal tumors biopsies as it provides an embryonic niche well suited for *in vivo* studies of neuroblastoma [64]. In addition, mechanistic studies will be required to assess the functional role of the genes exposed in this study. We have previously reported the detailed characterization of the OCCC-derived cancer stem cell subpopulations as ovarian CSC (5) and the extensive variation of gene expression and epigenetic changes together with variation at the level of chromosomal changes and SNP chip analysis, which strongly suggest that both cancer stem cells and clonal evolution mutually contribute to intratumoral heterogeneity. Our results further emphasize the urgent need for developing novel anti-cancer therapeutic approaches that will take into consideration intratumoral heterogeneity of cancer cells including that of the cancer stem cell subpopulations.

## Supporting Information

Materials and Methods S1 **RNA extraction from laser micro-dissected tumor samples, and from *in vitro* growing cells, qRT-PCR analyses and Bisulfite sequencing are described.** (DOCX)

Figure S1 **Ingenuity Pathway analysis (IPA).** Illustration of WNT signaling pathway related genes and their altered expression in CCSPs C12 and C13 –derived tumors generated i.m and i.t.
(TIF)

Figure S2 **Ingenuity Pathway analysis (IPA).** Illustration of Notch signaling pathway related genes and their altered expression in CCSPs C12 and C13 –derived tumors generated i.m and i.t.
(TIF)

Figure S3 **Ingenuity Pathway analysis (IPA).** Illustration of Hedgehog signaling pathway related genes and their altered expression in CCSPs C12 and C13 –derived tumors generated i.m and i.t.
(TIF)

Table S1 **Enriched GO terms in C12 and C13 – derived tumors generated i.m and i.t.**
(DOCX)

Table S2 **Primers used for Quantitative real time RT-PCR.**
(DOCX)

Table S3 **Primers used for bisulfite sequencing.**
(DOCX)
