# Supplementary material for: Niche-Dependent Gene Expression Profile of Intratumoral Heterogeneous Ovarian Cancer Stem Cell Populations
Source: PLoS One. 2013 Dec 17;8(12):e83651. doi: 10.1371/journal.pone.0083651 (PMC3866276; doi:10.1371/journal.pone.0083651)
Supplement: Materials and Methods S1 — RNA extraction from laser micro-dissected tumor samples, and from in vitro growing cells, qRT-PCR analyses and Bisulfite sequencing are described. (DOCX) [file pone.0083651.s001.docx]

**Material and methods S1**

**RNA extraction from laser micro-dissected tumor samples, and from *in vitro* growing cells**

Harvested CCSP C12 and C13 - derived tumors (intramuscular (i.m) tumors) and teratomas bearing tumors (intrateratoma (i.t) tumors) were snap frozen in liquid nitrogen. 20µM cryo-sections were mounted on PALM MembraneSlides (Carl Zeiss), and the tumor cell morphologies were identified using cresyl violet staining (Sigma). Laser microdissection and pressure catapulting (LMPC) samples of identified tumor tissues were collected using the Palm® MicroLaser system (Palm, Germany). LMPC samples and *in vitro* grown cells were subjected to total RNA extraction procedures using the Qiagen RNeasy®Micro Kit and RNeasy®Mini Kit (respectively). RNA samples were as follows: CCSP C12 *in vitro* grown cells, i.m tumor and i.t tumor, and CCSP C13 *in vitro* grown cells, i.m tumor and i.t tumor. The quality of the RNA samples was analyzed using the LabChip microfluidic technology with the Experion system (BioRad Laboratories).

**qRT-PCR analyses**

One-step qRT-PCR analysis was performed using the QuantiTect SYBRGreen RT-PCR kit (Qiagen,Inc.). Total RNA (40 ng) was converted into cDNA and subjected to 25-40 PCR cycles. Expression measurement of the genes of interest was performed using the Rotor-Gene RG-3000A system (Corbett Research), and analyzed by using the Rotor-Gene software (Corbett Research). The relative gene expression values were normalized using β-actin and GAPDH as independent internal control genes, and calculated by the ∆∆CT method. The N-fold differential expression of the evaluated genes for CCSP C12 compared with CCSP C13 was calibrated by averaging the results obtained for each gene normalized against both β-actin, and GAPDH. The primers used for gene amplification of the genes described in this study are provided in Supplemental Table S2.

**Bisulfite sequencing**

Genomic DNA (1 µg) was bisulfite-converted with the Methylamp DNA Modification kit (EPIGENTEK, NY) according to the manufacturer’s instructions. After bisulfite conversion, DNA was amplified using Faststart Taq polymerase (Roche). PCR products were TA-cloned into pGEM®T vector (Promega). Eight clones were examined for each gene and the inserts were sequenced with M13 universal primers. Primer sequences and annealing temperatures used for these analyses are provided in Supplemental Table S3.
