# Supplementary figures and images for: Niche-Dependent Gene Expression Profile of Intratumoral Heterogeneous Ovarian Cancer Stem Cell Populations
Source: PLoS One. 2013 Dec 17;8(12):e83651. doi: 10.1371/journal.pone.0083651 (PMC3866276; doi:10.1371/journal.pone.0083651)

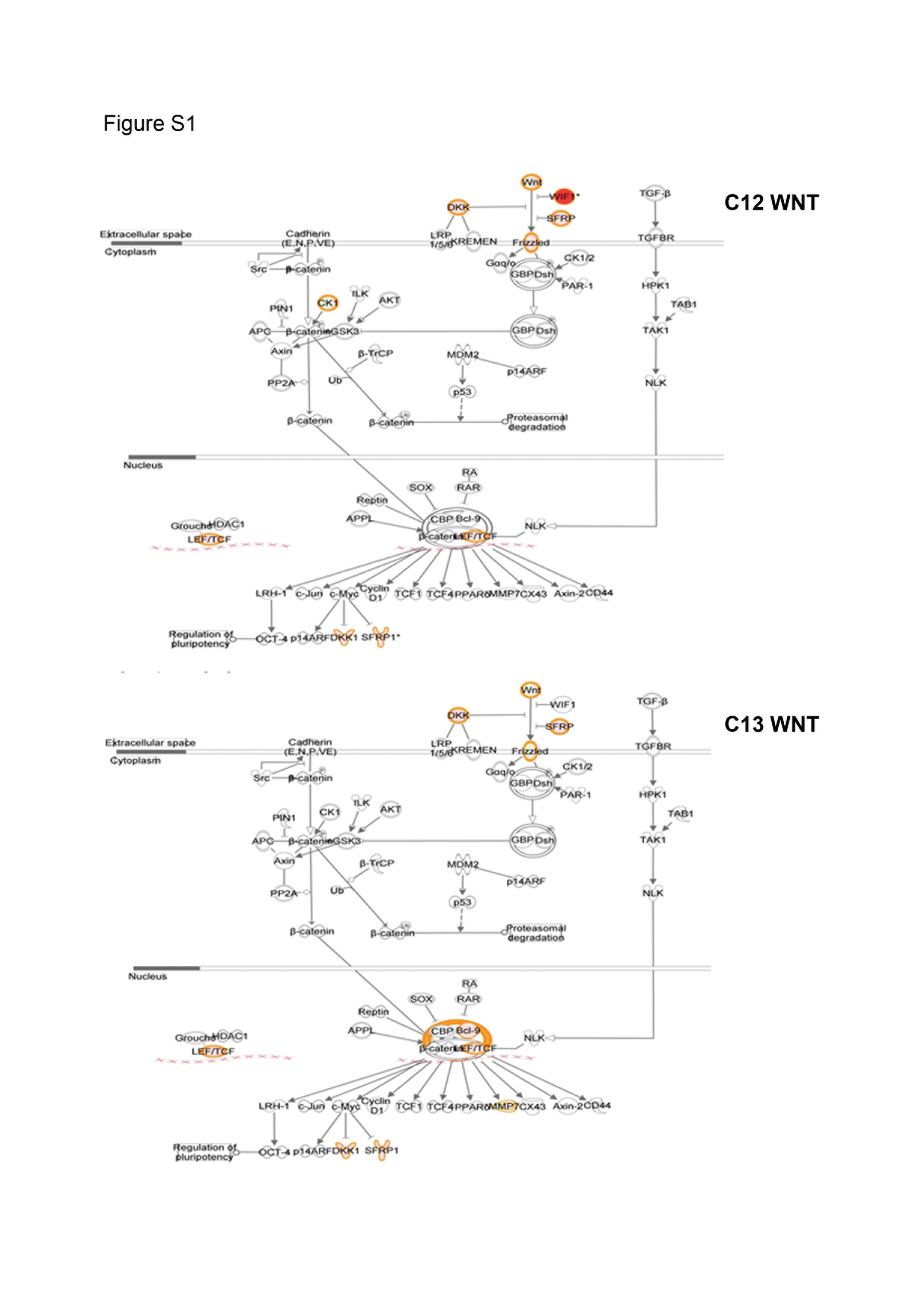

Supplement: Figure S1 — Ingenuity Pathway analysis (IPA). Illustration of WNT signaling pathway related genes and their altered expression in CCSPs C12 and C13 –derived tumors generated i.m and i.t. (TIF) [file pone.0083651.s002.tif]

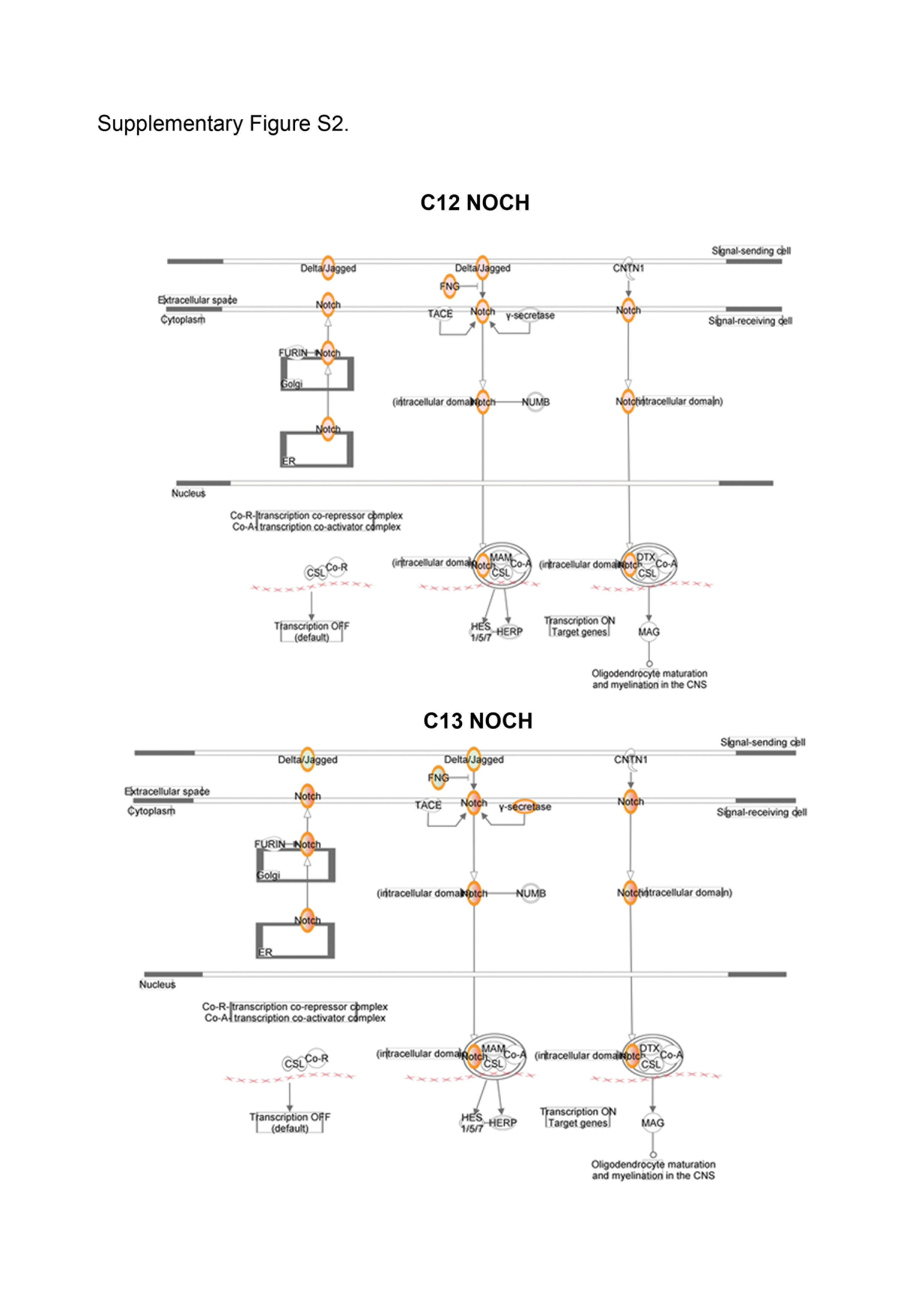

Supplement: Figure S2 — Ingenuity Pathway analysis (IPA). Illustration of Notch signaling pathway related genes and their altered expression in CCSPs C12 and C13 –derived tumors generated i.m and i.t. (TIF) [file pone.0083651.s003.tif]

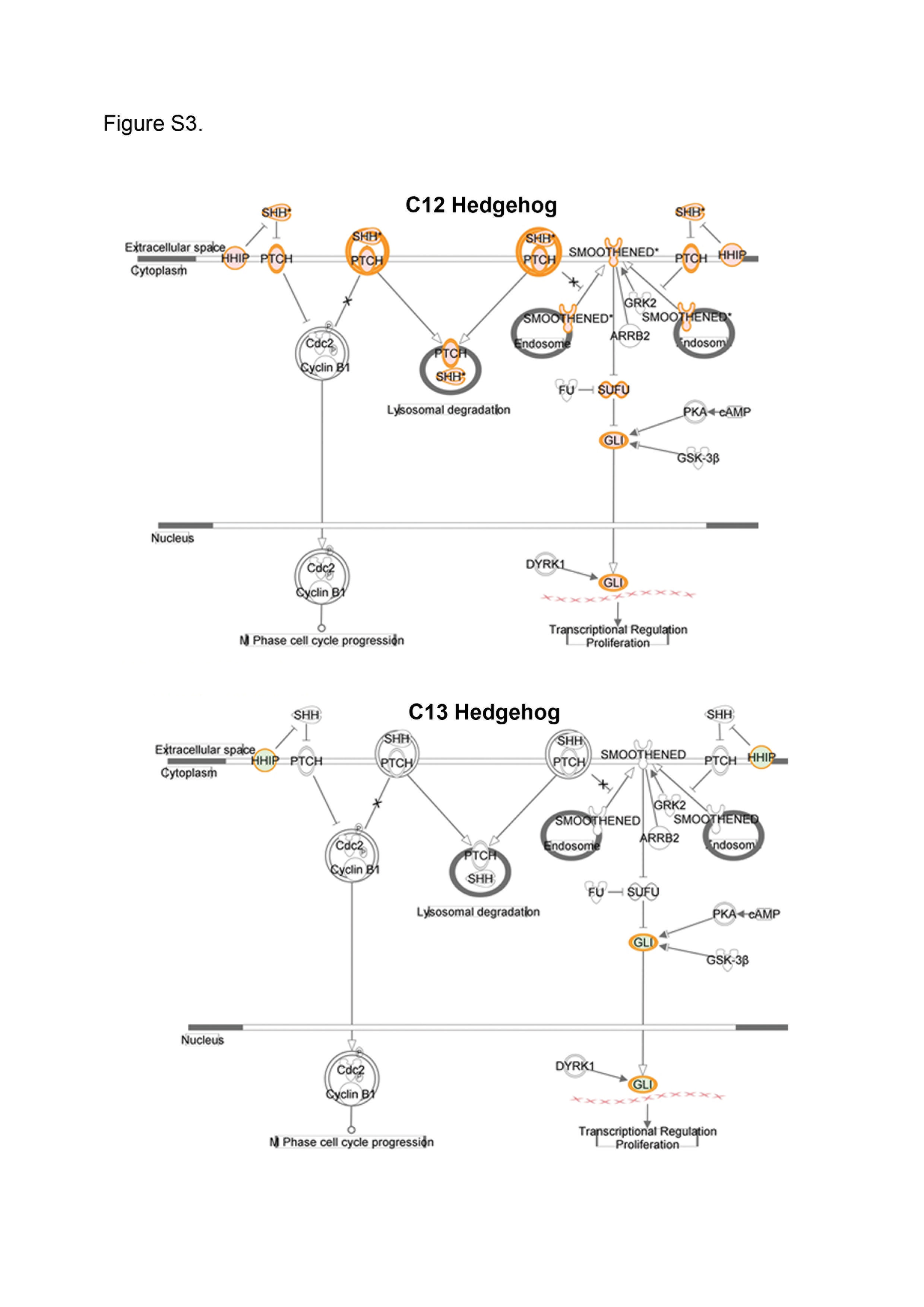

Supplement: Figure S3 — Ingenuity Pathway analysis (IPA). Illustration of Hedgehog signaling pathway related genes and their altered expression in CCSPs C12 and C13 –derived tumors generated i.m and i.t. (TIF) [file pone.0083651.s004.tif]
