# Supplementary material for: Niche-Dependent Gene Expression Profile of Intratumoral Heterogeneous Ovarian Cancer Stem Cell Populations
Source: PLoS One. 2013 Dec 17;8(12):e83651. doi: 10.1371/journal.pone.0083651 (PMC3866276; doi:10.1371/journal.pone.0083651)
Supplement: Table S1 — Enriched GO terms in C12 and C13 – derived tumors generated i.m and i.t. (DOCX) [file pone.0083651.s005.docx]

**Table S1.** **Enriched GO terms in C12 and C13 – derived tumors generated i.m and i.t.**
