# Supplementary material for: Niche-Dependent Gene Expression Profile of Intratumoral Heterogeneous Ovarian Cancer Stem Cell Populations
Source: PLoS One. 2013 Dec 17;8(12):e83651. doi: 10.1371/journal.pone.0083651 (PMC3866276; doi:10.1371/journal.pone.0083651)
Supplement: Table S2 — Primers used for Quantitative real time RT-PCR. (DOCX) [file pone.0083651.s006.docx]

| Gene Symbol | Position | Forward primer | Reverse primer |
| --- | --- | --- | --- |
| ABCC3 | 1780-1962 | 5’- GGCCTTTGTGTCTGTGTCCTTG -3’ | 5’- TGATGGCATAGCCTGGGGAGAT -3’ |
| ALDH1L1 | 2746-2854 | 5’- GCCTGGCTTCTGGTGTCTTC -3’ | 5’- GCCACGTCGGTCTTGTTGTA -3’ |
| β-ACTIN | 1041-1184 | 5’- CCTGGCACCCAGCACAAT -3’ | 5’- GGGCCGGACTCGTCATACT -3’ |
| CYP3A5 | 1311-1550 | 5’- TGACCCAAAGTACTGGACAG -3’ | 5’- TGAAGAAGTCCTTGCGTGTC -3’ |
| DKK3 | 26-189 | 5’- TGTGCTTGGTCCACCCCAGGTA -3’ | 5’- AGCAACCGAACCCGGATCCT -3’ |
| GALC | 308-562 | 5’- CTCCCGACTTCTAGTAAATTACC -3’ | 5’- GACCATGGCAACCCAATGAGTG -3’ |
| GAPDH | 175-282 | 5’- ATGGGGAAGGTGAAGGTCG -3’ | 5’- GGGGTCATTGATGGCAACAATA -3’ |
| GPX3 | 708-907 | 5’- GTACATCTGACCGCCTCTTCTG -3’ | 5’- GGCCTTCAGTTACTTCCTCTTG -3’ |
| KISS1R | 393-539 | 5’- AACTTCTACATCGCCAACCTG -3’ | 5’- CACCGAGACCTGCTGGATGTA -3’ |
| LCN2 | 330-571 | 5’- TCACCTCCGTCCTGTTTAGG -3’ | 5’- CGAAGTCAGCTCCTTGGTTC -3’ |
| MX1 | 1665-1945 | 5’- GACAGGACCATCGGAATCTT -3’ | 5’- GTGATGAGCTCGCTGGTAA -3’ |
| TACSTD2 | 1755-1857 | 5’- TACCCGAGGAGAAGAGGAGTTT -3’ | 5’- AGGCTTCTTTCCCAGTGACAAG -3’ |

**Table S2: Primers used for Quantitative real time RT-PCR**
