# Supplementary material for: Niche-Dependent Gene Expression Profile of Intratumoral Heterogeneous Ovarian Cancer Stem Cell Populations
Source: PLoS One. 2013 Dec 17;8(12):e83651. doi: 10.1371/journal.pone.0083651 (PMC3866276; doi:10.1371/journal.pone.0083651)
Supplement: Table S3 — Primers used for bisulfite sequencing. (DOCX) [file pone.0083651.s007.docx]

| Gene Symbol | Position | Forward primer | Reverse primer |
| --- | --- | --- | --- |
| GPX3 | -207-13 | 5’- GGAGTTAAAAGAGGAAGGG -3’ | 5’- CCCAACCACCTTTCAAAC -3’ |
| KISS1R | -15-185 | 5’- GGAAGGTAAGGTTAGGGGTGG -3’ | 5’- TCCAAAATTACRCCCTAACAC -3’ |
| MX1 | 5414-5654 | 5’- GGATATGTTTAGGTTTAAGG -3’ | 5’- CTCACAAACCCTATACTAA -3’ |
| TACSTD2 | -30-168 | 5’- TTAGGTTTGTAGTAGGAGGT -3’ | 5’- CTAATATTTAAATAACACATCC -3’ |

**Table S3: Primers used for bisulfite sequencing**
